# Effect of curcumin and *Garcinia kola* on the cerebellum of the transected sciatic nerve of the diabetic rats: glial and neuronal evidence

**DOI:** 10.3389/fnins.2026.1758544

**Published:** 2026-02-16

**Authors:** Abdullah Hilmi Marangoz, Hala Mahgoub Hamour, Arife Ahsen Kaplan, Işınsu Alkan, Gamze Altun, Süleyman Kaplan

**Affiliations:** 1 Department of Neurosurgery, Faculty of Medicine, Ondokuz Mayis University, Samsun, Türkiye; 2 Department of Histology and Embryology, Faculty of Medicine, Ondokuz Mayis University, Samsun, Türkiye; 3 Department of Histology and Embryology, Faculty of Medicine, Bolu Abant Izzet Baysal University, Bolu, Türkiye; 4 Department of Basic Sciences, Faculty of Dentistry, Nevşehir Haci Bektaşi Veli University, Nevşehir, Türkiye

**Keywords:** apoptosis, curcumin, diabetes mellitus, *Garcinia kola*, glial activation, volume fraction

## Abstract

**Objective:**

Peripheral nerve injury and diabetes can lead to some serious neurodegenerative changes in central nervous system structures. Curcumin and *Garcinia kola* are considered natural compounds with known antioxidant and neuroprotective properties. The current study aims to investigate the effects of curcumin and *Garcinia kola* on granular cells, glial cells, and Purkinje cells in a diabetic rat model induced by sciatic nerve transection, using stereological and immunohistochemical approaches.

**Method:**

Thirty-five male adult rats were divided into five groups: Control, Sham (Transected Sciatic Nerve), Transected Sciatic Nerve + Diabetes (T + DM), T + DM + Curcumin (T + DM + Cur), and T + DM + *Garcinia kola* (T + DM + GK). Ninety days after the sciatic nerve injury, the rats' cerebellar tissues were dissected and processed into paraffin and resin blocks. Volumetric fractions of cortical layers and white matter were estimated using the Cavalieri principle. Apoptosis and glial activation were assessed by immunohistochemical analysis.

**Results:**

Stereological analyses revealed that the volume fraction of the molecular layer was significantly lower in the T + DM + Cur group compared to the T + DM + GK and T + DM groups. A significant decrease in the volume fraction of the Purkinje cell layers was observed in the T + DM + Cur group compared to the Sham group. No significant differences were found between the groups in the granular layer and white matter volume fractions. Immunohistochemical analysis revealed inhibition of caspase-3 activity in the T + DM group. However, intense caspase-3 activation was also observed in the Control and T + DM + Cur groups.

**Conclusion:**

Sciatic nerve transection, combined with diabetes, leads to significant structural and cellular changes in the cerebellar cortex. The mechanisms underlying the anti-apoptotic effects of diabetes on granular cells and other cerebellar components should be investigated in detail.

## 1 Introduction

Diabetes mellitus (Sullivan et al., 2008) is a metabolic and chronic disease that affects the central nervous system (Oh et al., 2021). Cognitive problems are among the most common central nervous system disorders due to Type 1 and Type 2 DM (Zilliox et al., 2016). It is known that insulin receptors are more densely concentrated in the cerebellum than in the rest of the brain. However, the relationship between cerebellar changes, insulin resistance, and cognitive and emotional changes is not fully understood (Zhang et al., 2024). It has been previously reported that gray matter volume in the cerebellum decreases in type 2 DM (Oh et al., 2021). Neuroimaging studies have reported abnormal neural activity in the cerebellum of patients with DM in regions that play a key role in cognitive functions (He et al., 2025). Based on the above studies, interest in the effects of diabetes on the cerebellum has been growing in recent years. The cerebellum's role in motor and cognitive functions makes this effect and the resulting treatment options valuable. Anatomical studies have revealed that the cerebellum plays a key role not only in motor function but also in cognitive function (Buckner, 2013). The cerebellum is the target of many sporadic and hereditary neurodegenerative diseases. In particular, Purkinje cells have been reported to be sensitive to hypoxia but resistant to hypoglycemia (Koeppen, 2018). Therefore, in explaining the functional and morphological relationships of diabetes on the central nervous system, it is essential to reveal the changes in histological structures in the cerebellum at the cellular and protein levels.

Phytotherapeutic approaches are being developed to combat the neurodegeneration and neuroinflammation that occur due to DM. In this context, the antioxidant and anti-inflammatory properties, as well as the neuroprotective activity, of curcumin, the active polyphenolic compound of *Curcuma longa*, and *Garcinia kola* have been reported in the literature (Azzini et al., 2024; Seke Etet et al., 2022). Evidence has been presented that Curcumin in diabetes reduces macrophage infiltration and neuronal damage by suppressing the proinflammatory process, thereby reducing the accumulation of reactive oxygen species (ROS; Islam et al., 2025; Soetikno et al., 2011). Similarly, *Garcinia kola*, used therapeutically in a diabetic rat model, was observed to protect Purkinje cells by suppressing apoptosis and neuroinflammation in cerebellar tissue (Farahna et al., 2017).

It has been reported that peripheral nerve injuries can affect the central nervous system and local loss of function (Vaudano et al., 1998). Peripheral neural changes can lead to various effects on the central nervous system. Animal studies have shown that surgical transposition of the sciatic nerve changes brain function (Yuan et al., 2022). In this context, it is essential to examine the neuronal effects of sciatic nerve injury on the cerebellum in diabetic rats. Based on this, our study aims to investigate the neuroprotective effects of curcumin and *Garcinia kola* against potential cerebellar degeneration in diabetic brains following sciatic nerve transection. We immunohistochemically assessed the impact of astroglia activation and apoptosis, and how these phytotherapeutic agents may modify neuroinflammation in the cerebellum resulting from diabetes and peripheral neuropathy. The current study examines responses at the neuronal and glial levels in the cerebellum of diabetic rats using a surgical nerve transection model.

## 2 Materials and methods

### 2.1 Animals and ethical approval

All surgical and experimental procedures related to our study were conducted in accordance with the highest standards of animal welfare and ethics. The Ondokuz Mayis University Animal Experiments Local Ethics Committee approved the experimental and surgical procedures of the study. Cerebellar cadaveric tissues in our study were obtained from experimental research dated October 17, 2019, and decision number 2019/49, and permission was received from the Ondokuz Mayis University Animal Experiments Local Ethics Committee (Petition no: E-68489742-604.02-2500247633, date: October 30, 2025). All surgical and experimental procedures were performed in accordance with ARRIVE and the Guide for the Care and Use of Laboratory Animals.

**Figure 1:**
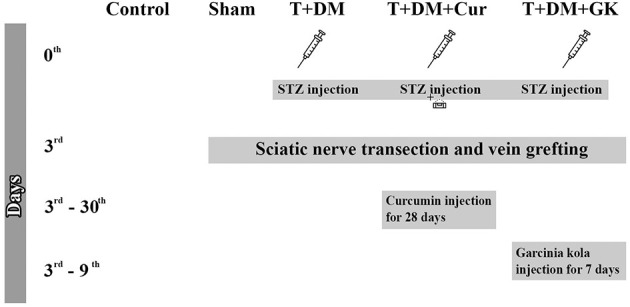
Timelines of the groups.

In this study, 35 male Wistar albino rats (12 weeks old, 250–300 g) were used. These rats were divided into five groups, each consisting of seven animals. All rats were kept in separate cages. The control group (Fernandez-Castaneda et al., 2022) was used to obtain baseline values. No procedure was performed in this group. In the sham group, vein grafting was performed in non-diabetic rats after sciatic nerve transection. A 10-mm section was cut with microscissors 2 cm distal to the sciatic nerve notch, and vein grafts were sutured to the nerve ends. A single dose of 50 mg/kg 0.1 M streptozotocin (STZ; dissolved with freshly prepared citrate buffer, pH: 4.5) was intraperitoneally given to rats in the Transected Nerve + Diabetic Group (T + DM). After establishing a diabetic model in this group, sciatic nerve transection and vein grafting were performed. After creating a diabetic model and a transected sciatic nerve and vein graft model in the Transected + Diabetes Mellitus + Curcumin Group (T + DM + Cur), 300 mg/kg/day curcumin was given by intragastric gavage for 28 days. The rats in the Transected + Diabetes Mellitus + *Garcinia kola* Group (T + DM + GK) received 200 mg/kg/day *Garcinia kola* by intragastric gavage for 7 days after diabetic model induction, followed by a transected sciatic nerve and vein graft model. All antioxidants were administered between 9:00 and 10:00 a.m. Rats in all groups were sacrificed 90 days after the experiment began (Deniz et al., 2025; Denizci et al., 2024; Hamour et al., 2025; Figure 1).

### 2.2 Surgical procedures

All surgical procedures and evaluations were performed under deep sedation or general anesthesia. Rats were anesthetized with 50 mg/kg ketamine (Ketalar^®^, Eczacibaşi, Istanbul, Turkey) and 10 mg/kg xylazine (Rompun^®^, Bayer, Istanbul, Turkey) administered intraperitoneally (i.p.; Hamour et al., 2025; Onger et al., 2023). After general anesthesia was achieved, surgery was performed to remove hair from the neck and groin areas, and the area was then disinfected (using a 0.5% chlorhexidine solution prepared in 70% alcohol). The effectiveness of the anesthesia was assessed by checking the finger-squeeze reflex. The vein graft to be used for sciatic nerve repair was isolated from the external jugular vein. A segment of vein measuring approximately 11.3 mm in length and 2.1 mm in diameter was removed, and the cut ends were sutured to ensure hemostasis. The vein graft was irrigated with physiological saline and prepared for usef. To transect the sciatic nerve, the gluteal muscles were removed to expose the sciatic nerve. A 10-mm segment of the nerve was dissected approximately 2 cm distal to the sciatic notch. The vein graft was then irrigated with physiological saline and anastomosed into the approximately 3 mm epineural sheath of the proximal and distal nerve ends using 10-0 atraumatic nylon suture (Ethicon EH 7438G, Germany). At the end of the surgical procedures, cefazolin sodium (Equizoline IM/IV) was administered at doses of 4, 2, and 1 mg/kg, respectively, for three postoperative days to reduce the risk of postoperative infection. Antibacterial spray (Pederipra, Hipra, Spain) was also applied (Hamour et al., 2025). All surgical procedures and termination of experiments were performed in accordance with ethical guidelines. At the end of the experiment, animals were anesthetized intraperitoneally with a combination of ketamine (50 mg/kg, Ketalar^®^, Eczacibaşi, Istanbul, Turkey) and xylazine (10 mg/kg, Rompun^®^, Bayer, Istanbul, Turkey; i.p.). After the complete loss of pedal reflexes confirmed deep anesthesia, the animals were euthanized with high-dose anesthetic administration, followed immediately by cardiac perfusion. The animals were secured in the supine position on a dissection board. The abdominal wall was opened, the rib cage was accessed, and the sternum was elevated to fully expose the heart. A cannula was carefully placed at the apex of the left ventricle, and a small incision was made in the right atrium to ensure perfusion outflow. Initially, a 0.9% isotonic sodium chloride solution was administered to clear blood from the systemic circulation. Following this, a 4% paraformaldehyde-2% glutaraldehyde solution was perfused to fix the tissue. Perfusion effectiveness was monitored throughout the perfusion process by color changes in the organs and tissues (Gage et al., 2012; Wu et al., 2021). Cerebellar tissues were dissected and placed for post-fixation. Following fixation, the tissue was subjected to electron and light microscopy.

### 2.3 Paraffin and resin blocks

Dissected cerebellar tissues were processed for paraffin and resin embedding for light and electron microscopic examination, respectively. To prepare the paraffin blocks, tissue samples were stored in a fixative containing 4% paraformaldehyde and 2% glutaraldehyde, passed through an ascending alcohol series, and cleared with xylene. The tissues were then infiltrated in paraffin at 58 °C for 3 h in an oven and embedded in paraffin blocks. Code numbers were assigned to the paraffin block to ensure that all analyses were conducted blindly. Serial 5-μm-thick sections were cut from these paraffin blocks using systematic random sampling principles (Leica RM2245 microtome, Leica, Nussloch, Germany) for use in stereological analyses. Sections were stained with hematoxylin and eosin. Additional 4-μm-thick sections for immunohistochemical staining were taken from the same blocks. Additionally, one cerebellar sample from each group was taken for electron microscopic examination to prepare resin blocks for further analysis. Histopathological evaluation was performed by taking 500-nm-thick sections from the resin blocks and staining them with toluidine blue.

### 2.4 Volumetric fractions using Cavalieri's method

**Figure 2:**
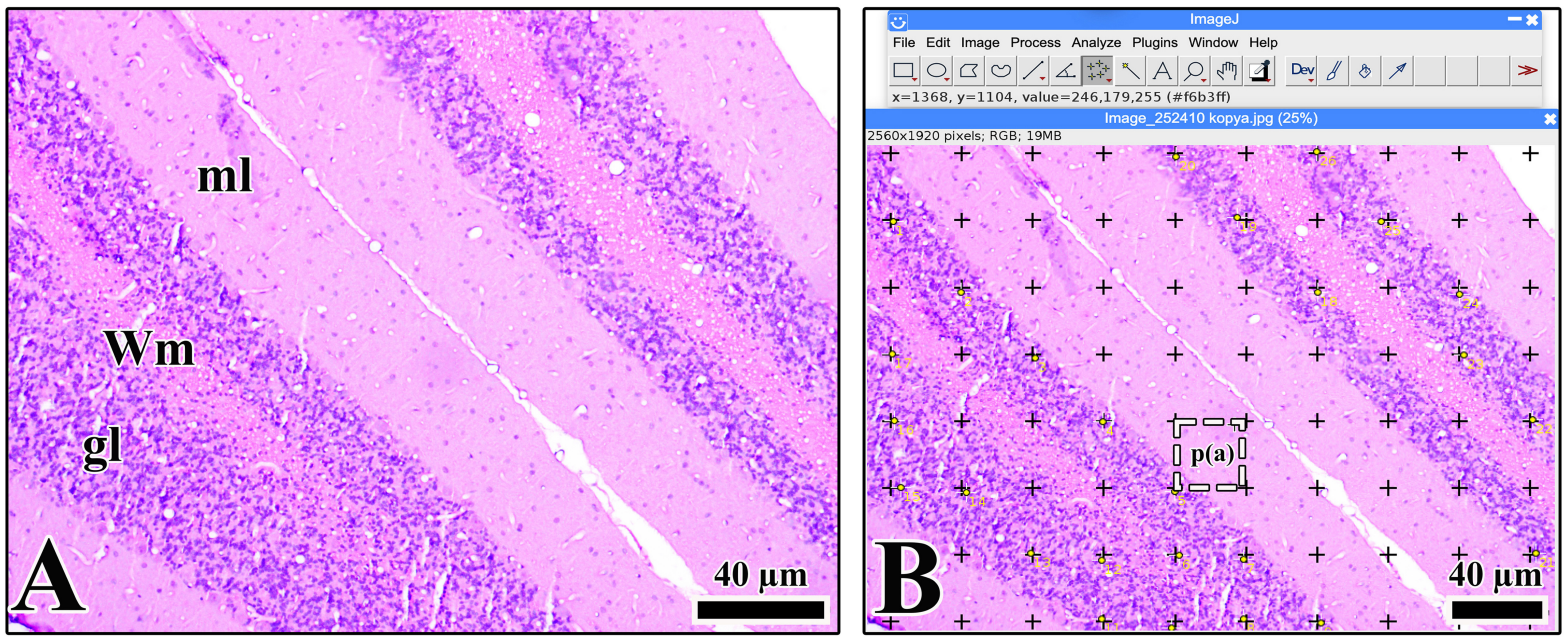
**(A)** Light microscopic image of a cerebellar section stained with hematoxylin-eosin. **(B)** Stereological analysis of the volume fraction of the granular layer of the cerebellum using the ImageJ program. The area represented by a point is indicated by p(a). gl, granular layer; ml, molecular layer; Wm, white matter.

A point counting method based on the Cavalieri principle was used (Cruz-Orive, 1999; Gundersen and Jensen, 1987; Altunkaynak et al., 2009) to calculate the volume fractions of the cerebellar cortex layers. This method used serial slice images obtained through systematic random sampling using ImageJ software. A test grid of equally spaced points was placed on each slice. Each anatomical region, including the molecular layer, Purkinje cell layer, granular layer, and white matter, was assessed according to areas on the grid. Percentages were obtained by dividing the number of points in each layer by the total number of points, and the volume fraction was calculated (Figure 2).

### 2.5 Immunohistochemical analyses

Four μm-thick sections from paraffin blocks were placed on positively charged slides and deparaffinized in an oven at 60 °C. After deparaffinization, the sections were passed through a xylol series and a gradually decreasing alcohol series. After incubation in 3% hydrogen peroxide to eliminate endogenous peroxidase activity, the sections were treated with citrate buffer (pH 6) at 800 W for 3 min, followed by 170 W for 17 min for antigen retrieval. Protein blocking solution was then applied to prevent nonspecific staining (Altun and Kaplan, 2025). Following these steps, the sections were incubated overnight at +4 °C with anti-caspase-3 (dilution: 1:200, ab4051, Abcam, RRID: AB 304243) and anti-GFAP (dilution: 1:200, MAB360, Sigma, RRID: AB_11212597) as the primary antibodies used in the study. After primary antibody incubation, the sections were treated with a biotinylated secondary antibody and a streptavidin-peroxidase complex. AEC chromogen (Abcam, USA) was used to visualize the reaction product. Red-brown stained areas were considered positive. Immunohistochemical staining was performed using the Mouse Specific HRP/AEC detection kit (ab93705, Abcam, USA). Counterstaining was used with Mayer's hematoxylin. Staining intensity was assessed qualitatively.

### 2.6 Statistical analyses

Statistical analyses were performed using GraphPad Prism (version 10.5.0). In evaluating the statistical data obtained from stereological analyses, the Shapiro-Wilk normality test was first performed to determine the distributional characteristics of the groups. One-Way ANOVA (*Post-hoc* Tukey test) was applied to groups with normal distribution, and Kruskal-Wallis (*Post-hoc* Dunn's test) was applied to groups with non-normal distribution. In all statistical analyses, *p* < 0.05 was considered significant. Results were expressed as mean ± standard deviation.

## 3 Results

### 3.1 Stereological data

**Figure 3:**
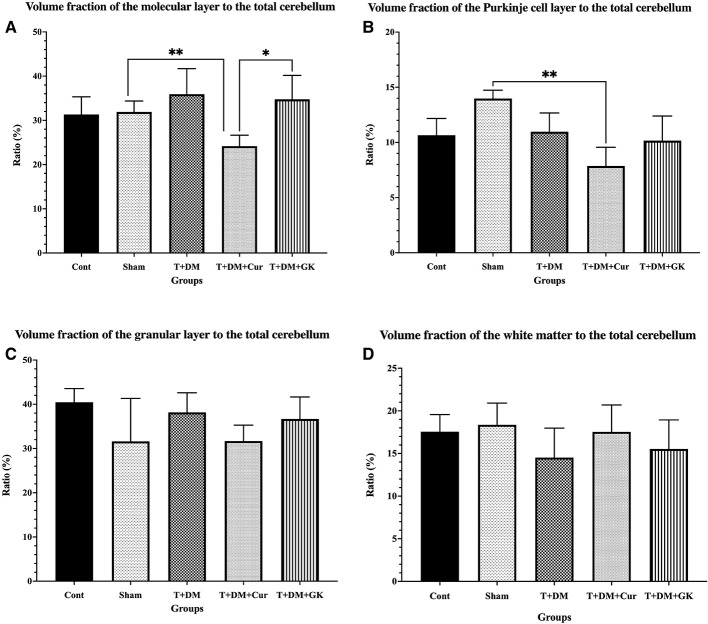
The volume fractions of cerebellar layers in all groups of the total cerebellum volume are shown. **(A)** When examined in terms of molecular layer volume fraction, the volume fraction in the T + DM + CUR group decreased compared to the T-DM group (**p < 0.01). Similarly, the volume fraction in the T + DM + CUR group decreased compared to the T + DM + GK group (*p < 0.05). **(B)** When examining the volume fractions of the Purkinje cell layer, a significant decrease was observed in the T + DM + CUR group compared to the Sham group (**p < 0.01). **(C, D)** No significant difference was observed between the groups in the granular layer and white matter volume fractions of the cerebellum (*p* > 0.05). Values are presented as mean ± standard deviation (SD).

When the volume fractions of the cerebellar layers data were evaluated, no statistically significant difference was observed between the groups in terms of the ratios of white matter and granular layer volumes. However, when the volume changes in the molecular layer were evaluated, a statistically significant decrease was observed in the T + DM + Cur group compared to the T + DM and T + DM + GK groups (*p* < 0.01 and *p* < 0.05, respectively). Furthermore, when the Purkinje layer volume ratios were examined, a decrease was observed in the T + DM + Cur group compared to the Sham group (*p* < 0.05; Figure 3). Coefficient of error (CE) and coefficient of variation (CV) values for the stereological data are presented in Table 1.

**Table 1:** The coefficient of errors (CE) and the coefficient of variation (CV) for all groups.

**Groups**	The volume of the molecular layer		The volume of the Purkinje layer		The volume of the granular layer		The volume of the white matter	
	**CE**	**CV**	**CE**	**CV**	**CE**	**CV**	**CE**	**CV**
Cont	0.03	0.10	0.05	0.13	0.03	0.07	0.04	0.10
Sham	0.04	0.07	0.06	0.03	0.04	0.28	0.05	0.12
T + DM	0.04	0.14	0.08	0.14	0.04	0.10	0.07	0.21
T + DM + Cur	0.04	0.09	0.07	0.19	0.04	0.10	0.05	0.16
T + DM + GK	0.04	0.14	0.08	0.20	0.04	0.12	0.06	0.20

### 3.2 Histopathological assessment

**Figure 4:**
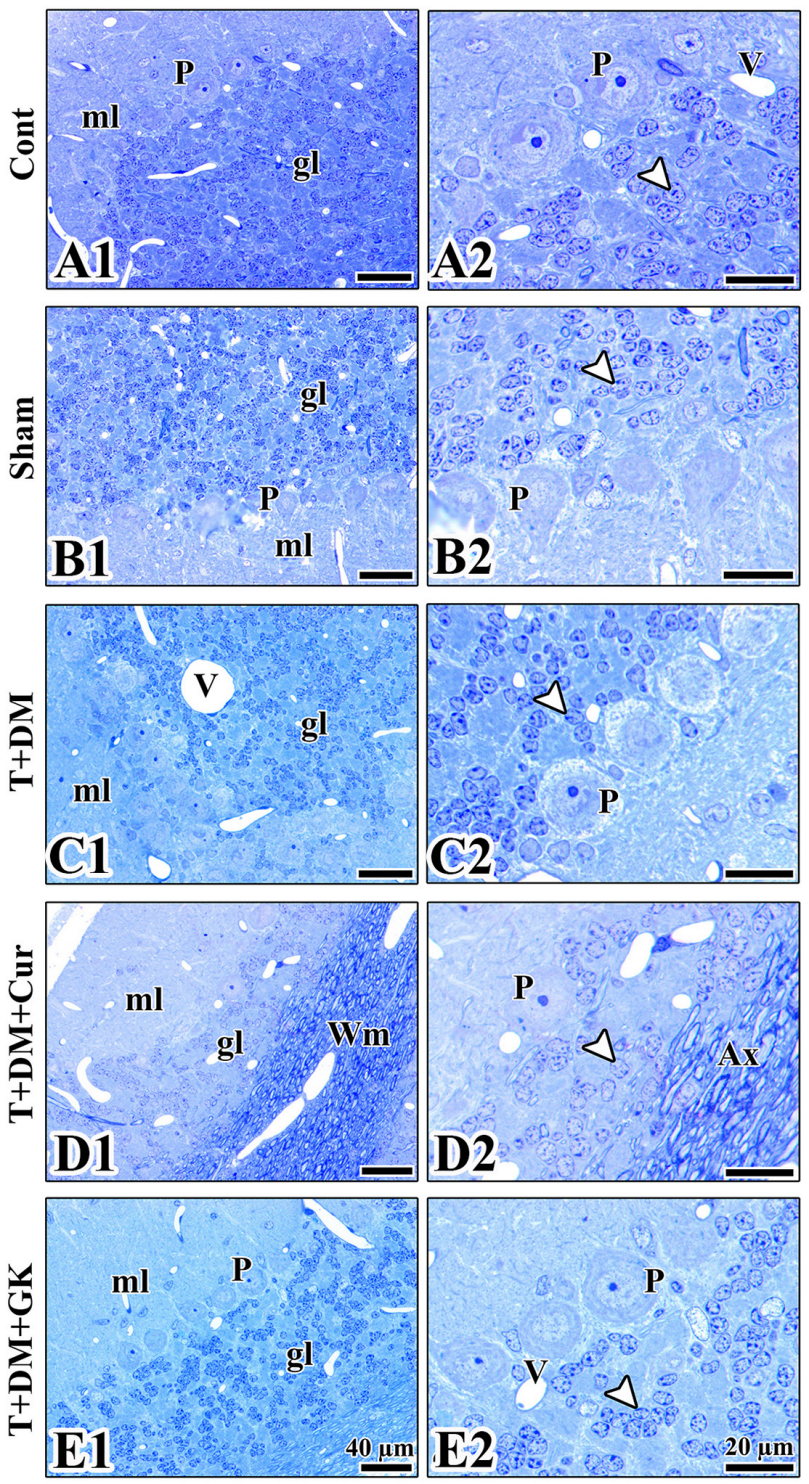
**(A1–E2)** The morphological structure of the granular layer and Purkinje layer of the cerebellum is observed in the images obtained from the resin blocks of the groups. **(A1, A2)** The cerebellar layers in the control group are observed to have a healthy morphology. The nuclear borders of the granular cells (arrowheads) and the nuclear and nucleolus borders of the Purkinje cells are observed to be distinct and healthy (P). **(B1, B2)** The cerebellar layers in the sham group are observed to have a normal structure. The pericellular space observed around the Purkinje layer in the sham group may be considered an artifact from tissue processing or an expansion due to edema. **(C1, C2)** In the T + DM group, the granular layer pattern is disrupted, and the size of the vessels and the distance between granular cells are increased. The prominent spaces around the perikaryon of Purkinje cells are a unique feature of this group. **(D1, D2)** In the T + DM + Cur group, more intense staining of the white matter (Wm) and a distinct structure of the axon bundles and an increased distance between granular cells (Ax) are observed. **(E1, E2)** A healthy cellular organization is observed in the granular and Purkinje layers in the cerebellar tissue of the T + DM + GK group. Each type of cell has clear borders and is easily differentiated from surrounding tissues. Arrowheads, granular cells; gl, granular layer; ml, molecular layer; P, Purkinje cell; V, Vessel. Bars: 40 μm **(A1, B1, C1, D1, E1)**, 20 μm **(A2, B2, C2, D2, E2)**. Cresyl violet staining, resin sections.

**Figure 5:**
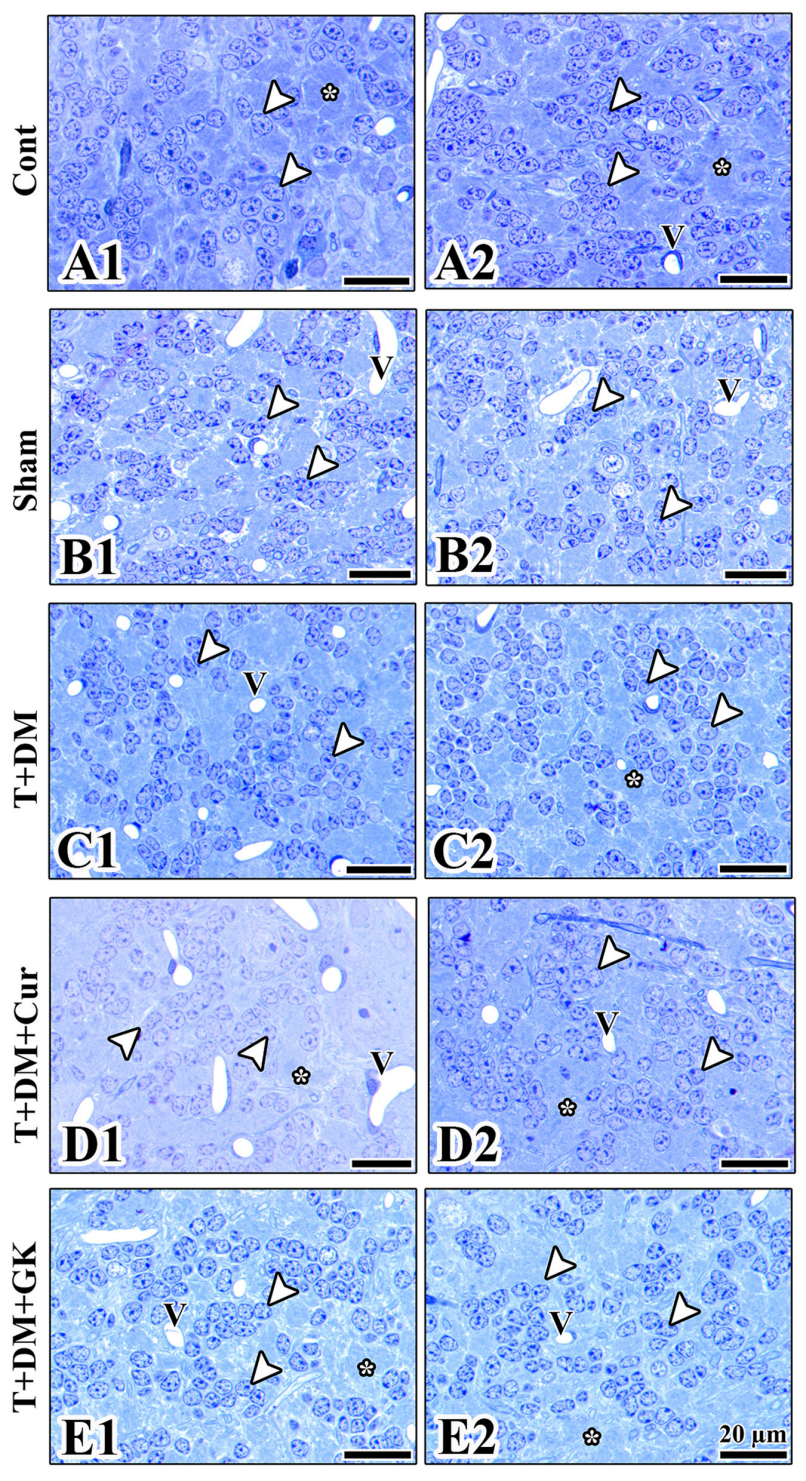
**(A1–E2)** The morphological structure of the granular layer of the cerebellum is observed in the images obtained from the resin blocks of the groups. **(A1, A2)** In the images of the control group, the granular cells (arrowhead) are observed to have normal morphology and are regularly distributed. **(B1, B2)** It is observed that the morphology of the granular cells (arrowhead) in the Sham group has a normal structure, but the distance between them was significantly increased. **(C1, C2)** In the T-DM group, an increased distance between granular cells was observed (arrowhead), and simultaneously, cytoplasmic paleness was noted in some cells. **(D1, D2)** In the T-DM-Cur group, a healthy structure is observed in the granular cells (arrowhead) after curcumin application; however, an increased distance between granular cells is also noted in this group. **(E1, E2)** It is observed that the granular layer maintains its cellular integrity in the T-DM-GK group; V, Vessel. Cresyl violet staining.

Histopathological examination of the cerebellar cortex revealed significant structural differences between the groups. In the control group, the healthy histological structure of the cerebellar layers was seen. Purkinje cells were single-layered, large, and had prominent nucleoli, while the dense structure of the granular layer was notable. In the sham group, slight disorganization was observed in the granular layer. Pericellular space formation was observed around the Purkinje cells. A slight expansion of the interstitial space was observed around some small clusters of granular cells. In the T + DM group, a decrease in granular layer cell density was observed due to diabetic damage. Mild sparsity of synaptic regions was observed in the molecular layer. In the T + DM + Cur group, curcumin treatment largely preserved the arrangement of the granular cell layer, but, in some areas of the Purkinje cell layer, it exhibited a decreased cell density. More intense staining of fiber bundles and axonal structures was observed in the white matter. Although the granular layer was generally preserved, morphological signs of healing and remodeling were noted. In the cells, the absence of damage progression was observed as a sign of healing. In particular, the cell boundaries were distinct. Degenerative findings such as nuclear pyknosis were not observed in this group. The prominent staining and more regular appearance of axonal extensions indicate axonal remodeling. In the T + DM + GK group, the granular layer's cellular integrity was mainly preserved, with Purkinje cells arranged in a regular pattern. Although vascular structures were prominent, tissue integrity was better preserved in this group (Figures 4, 5).

### 3.3 Immunopositive reaction of anti-glial fibrillary acidic protein

**Figure 6:**
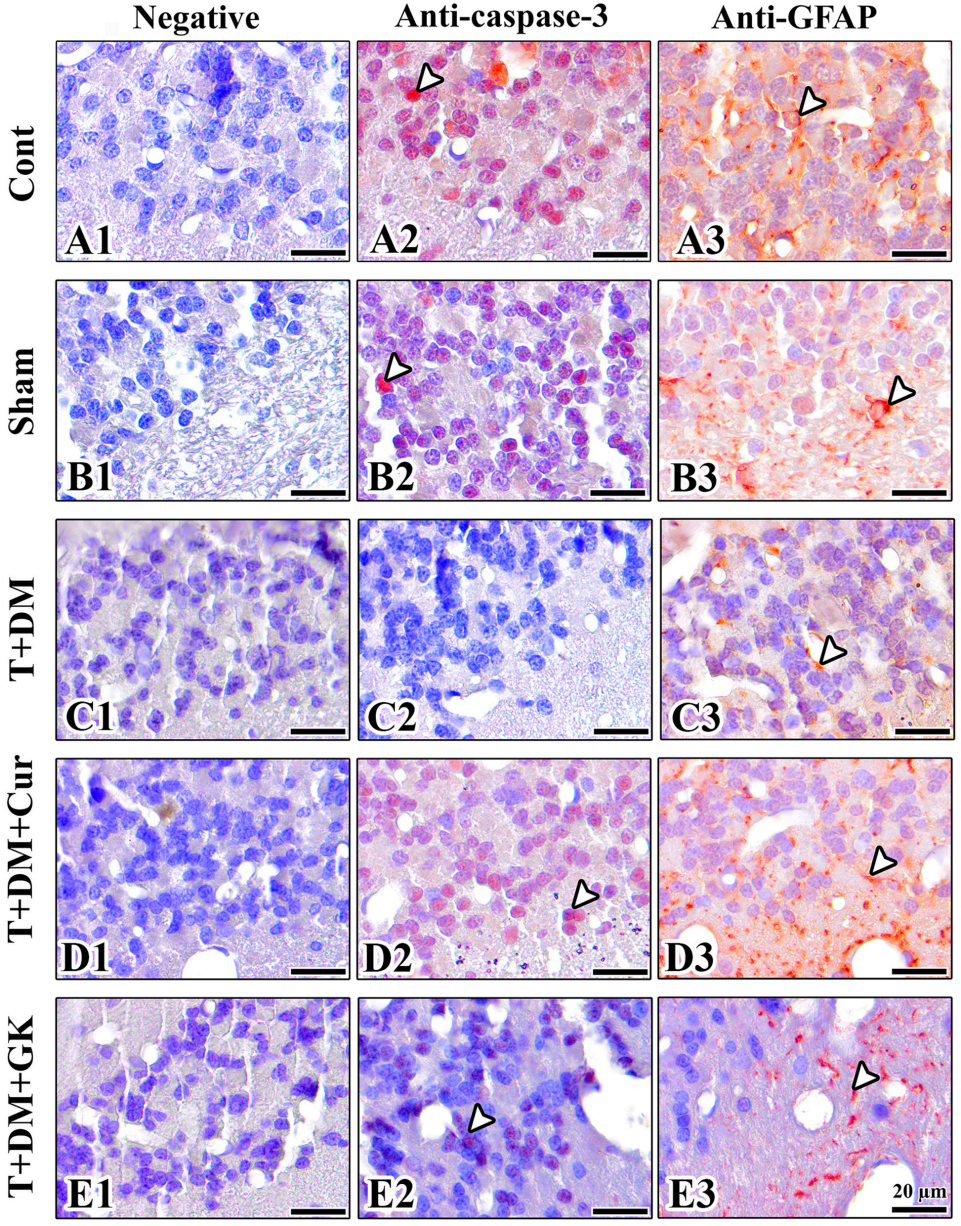
Immunohistochemical staining of the groups is shown. **(A1–E1)** Negative control images are shown in immunohistochemical analyses. **(A2–E3)** When all groups were evaluated for anti-caspase-3 and anti-GFAP activity, positive reactions are indicated by arrowheads. **(A2, A3)** Anti-GFAP and anti-caspase-3 activities were high in the control group. **(B2, B3)** Anti-GFAP and anti-caspase-3 activities were normal in the sham group. **(C2, C3)** In the T + DM group, anti-caspase-3 activity was inhibited and stained at low intensity, and anti-GFAP activity was also low. **(D2, D3)** In the T + DM + Cur group, an increase in anti-caspase-3 and anti-GFAP activity was observed compared to the T + DM group. **(E2, E3)** A decrease in anti-caspase-3 was observed in the T + DM + GK group compared to the Control and Sham groups. However, an increase in anti-GFAP immunoreactivity was observed in the T + DM + GK comparison with T + DM. Mayer's Hematoxylin was used for counterstaining.

When immunohistochemical staining from all groups was evaluated for GFAP positivity, intense GFAP expression was observed in the control group. Decreased GFAP activity was observed in the Sham group compared with the control group. GFAP positivity was observed at a low level in the T + DM group. Astrocytic reactivity was further increased in the T + DM + Cur group and the T + DM + GK group. However, immunoreactivity was weaker in the T + DM + GK group compared to the T + DM + Cur group (Figure 6).

### 3.4 Immunopositive reaction of anti-caspase-3

When immunohistochemically stained sections from all groups were evaluated for caspase-3 activity, intense caspase-3 expression was observed in the Control group. Caspase-3 activity was lower in the Sham group than in the Control group. No positive caspase-3 staining was observed in the DM group. An increase in cell immune reactivity was observed in the T + DM + Cur and T + DM + GK groups. The T + DM + GC and T + DM groups showed similar immunohistochemical intensities (Figure 6).

## 4 Discussion

The risk of developing cognitive disorders is higher in patients with type 2 DM (Moheet et al., 2015). The neurodegeneration process in all parts of the brain increases as the duration of type 2 DM disease increases (Antal et al., 2022). As is well known, the cerebellum also plays a significant role in cognitive and behavioral functions (Saeedi Borujeni et al., 2024; Lange et al., 2015). A previous study suggested that gestational diabetes reduces the layer thickness, cerebellar volume, and cell numbers of the cerebellar cortex after birth (Hami et al., 2016). Upon examining the data from our study, no differences were observed in the granular layer or the white matter of the cerebellum across all groups. However, unexpectedly, a decrease in volume was observed in the molecular layer of curcumin-treated diabetic rats compared with the DM and T + DM + GK groups. A decrease in volume fraction of the Purkinje cell layer was observed in the curcumin-treated group compared to the sham group.

It has been suggested that morphological changes in the cerebellum and pons occur as a result of chronic peripheral nerve injury. After long-term chronic peripheral nerve damage, adaptive neuronal plasticity and neurogenic effects can be observed in the hindbrain (Rusanescu and Mao, 2017). In diabetic rats, rapid cerebellar reorganization may have reduced damage. Thus, the T + DM group had no volumetric changes compared to the control and sham groups. The neurodegenerative effects of DM on the cerebellum may not have been fully reflected at the cellular level due to potential early morphological changes. Therefore, longer-term model studies in diabetic rats with sciatic nerve transection may be needed to understand the processes of reorganization and neurodegeneration. Histopathological findings indicate that curcumin and *Garcinia kola* provide protection at the cellular level. Both antioxidants played an active role in axonal remodeling and in preventing degenerative changes. Evidence regarding curcumin's role in the regulation of axonal structure is presented in the literature (Ma et al., 2016). Information in the literature regarding the effects of *Garcinia kola* on axon repair is limited.

Edema in the brain or cerebellum disrupts cell volume regulation. As is known, glial cells are located near blood vessels and nerve cell bodies. Therefore, they play a crucial role in maintaining ionic balance (Ochoa-de la Paz and Gulias-Canizo, 2022). Cerebellar glial cells may have been activated after sciatic nerve transection in diabetic rats, potentially leading to edema. The role of diabetes in causing glial edema in the central nervous system has been emphasized (Yang et al., 2025). However, the role of peripheral nerve damage in causing edema in the central nervous system is limited. Based on this, our study suggests that diabetes disrupts cerebellar fluid balance by impairing glial cell function. Furthermore, the reorganization resulting from sciatic nerve transection and vein grafting may have acutely triggered glial activity in the brain. Considering that this agent suppresses inflammation and, consequently, reduces intracellular fluid accumulation in the curcumin-treated groups, curcumin-induced volumetric reductions in the Purkinje and molecular layers may have occurred. The fact that GFAP expression in the curcumin group was similar to that in the control group provides evidence for this. This may be due to curcumin triggering glial activity by modulating the diabetes-induced neuronal stress response. Darkly stained glial cells may indicate increased glial protein content and reactive gliosis.

In this study, anti-caspase-3 immunoreactivity was reduced in the granular cell layer in the T-DM group. This suggests that apoptotic pathways are not triggered during the destructive process in granular cells of the cerebellum after diabetes, but rather that other pathways, such as endoplasmic reticulum or mitochondrial stress, are activated. Granular cells are strongly responsive to neurotrophic factors. In particular, BDNF can prevent cell death due to glucose deprivation in granular cells (Tong and Perez-Polo, 1998). On the other hand, it is known that insulin growth factor (IGF)-1 causes antiapoptotic effects during cerebellar development (Chrysis et al., 2001). IGF-1 plays a key role in the cell cycle progression of granular cell progenitor cells during cerebellar development (Werther et al., 1990). In our experimental model, sciatic nerve transection may have induced IGF-1 expression in the cerebellum. A previous study suggested that IGF-1 expression increases in Schwann cells after peripheral nerve injury (Sullivan et al., 2008). The overexpression of IGF-1 protects against apoptosis in cerebellar granular cells in both *in vitro* and *in vivo* models (Chrysis et al., 2001). In this context, the decrease in apoptotic activity in the Sham group compared to the Control group may be attributed to the increased antiapoptotic activity of IGF-1 resulting from nerve transection and vascular grafting. In the DM group, this inhibition of apoptotic activity may be due to neurons' resistance to diabetes, a result of chronic hyperglycemia-induced cellular stress. Hyperglycemia has been shown to increase neuronal stress and apoptosis, thereby promoting neuronal damage rather than resistance (Kumar et al., 2017). Similarly, decreased PI3K/Akt signaling pathway and increased apoptosis signals have been reported in neurons exposed to hyperglycemia; this suggests that hyperglycemia makes neurons more vulnerable to stress (Xiang et al., 2015). The discrepancy between our findings and the literature suggests that cerebellar neurons may have developed a region-specific response to chronic hyperglycemia. On the other hand, chronic hyperglycemia may trigger distinct death pathways in the cerebellum, such as ferroptosis or pyroptosis, rather than apoptosis. Further molecular studies are needed to investigate this resistance. Additionally, previous studies have demonstrated that diabetes triggers apoptosis via caspase-3, leading to neuronal damage in the pancreas and brain. Anti-apoptotic protein levels or cellular defense mechanisms may be more pronounced in the cerebellum than in other organs. Further studies are needed on this topic (Deniz et al., 2025; Denizci et al., 2024). Previous cancer studies have shown that specific components, such as garcinol from *Garcinia kola*, activate caspase-3 in cancer cells (Sewani-Rusike et al., 2016). However, *Garcinia kola* has generally been observed to suppress caspase-3 activation. Due to the lack of the tunnel staining method, a limiting step in our study, we were unable to obtain sufficient information on the apoptotic effect. Further studies are needed to explain the increase in caspase-3 expression in cells in the groups treated with *Garcinia kola* and curcumin.

Additionally, histopathological evaluation revealed no light microscopic changes in the granular cells of the T-DM group. On the other hand, the failure to demonstrate apoptotic activity using the TUNEL method and the inability to analyze IGF-1 expression in granular neurons and Purkinje cells are limitations of the presented study. Methodological approaches to examine IGF-1 and caspase-3 are essential for a clear understanding of the molecular pathways in the cerebellum of diabetic rats and the effects of antioxidant substances, including curcumin and *Garcinia kola*.

## 5 Conclusion

It was observed that sciatic nerve transection in a diabetic rat model resulted in various morphological changes in the cerebellum. Furthermore, the cerebellar remodulation resulting from sciatic nerve transection may have prevented the effects of diabetes from manifesting. Curcumin was more effective at regulating glial activity, as indicated by GFAP immunoreactivity, whereas *Garcinia kola* had limited effects. Furthermore, increased caspase-3 expression in the curcumin-treated group suggests the induction of adaptive stress mechanisms in the cerebellum, particularly in granular neurons. Inhibition of caspase-3 expression was also noted in the T + DM group, raising new questions about the reduced adaptive stress response capacity of granular neurons in T + DM. To further elaborate on the stereological and immunohistochemical results obtained in our study, a cellular investigation of apoptotic pathways in the cerebellum after T + DM is necessary.

## Data Availability

The raw data supporting the conclusions of this article will be made available by the authors, without undue reservation.
